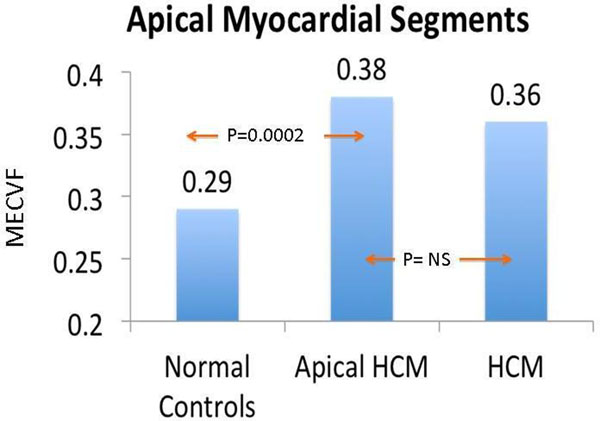# Quantitative of myocardial extracellular volume fraction improves characterization of fibrotic burden in patients with apical hypertrophic cardiomyopathy beyond visual assessment with late gadolinium enhancement

**DOI:** 10.1186/1532-429X-14-S1-O98

**Published:** 2012-02-01

**Authors:** Eri Watanabe, Yucheng Chen, Tomas G Neilan, Carolyn Ho, Ravi Shah, Ron Blankstein, Michael Jerosch-Herold, Raymond Kwong

**Affiliations:** 1 Brigham and Women's Hospital, Boston, MA, USA; 2 Radiology, Brigham and Women's Hospital, Boston, MA, USA

## Summary

Patients with apical hypertrophic cardiomyopathy (APH) have evidence of increased fibrotic burden, compared to normal controls, in myocardial segments without visible LGE. This finding may have diagnostic implication in patients with thickened apical segments of the LV and a clinical suspicion of APH.

## Background

Apical hypertrophic cardiomyopathy (APH), a variant of hypertrophic cardiomyopathy, is generally considered to have a benign clinical course, with localized hypertrophy and evidence of myofibril disarray and fibrosis at the apex alone. However, traditional late gadolinium enhancement (LGE) techniques are not sensitive enough to detect subclinical apical fibrosis. Accordingly, we hypothesized that quantitative myocardial extracellular collagen volume fraction (MECVF) via T1 mapping techniques at the apex would differentiate patients with and without APH.

## Methods

We performed 3T cardiac MRI in 35 subjects including 11 patients with APH including 3 patients without LGE and 8 with LGE (mean age 48 ± 7 years, all male, diagnosed via standard clinical and echocardiographic assessment), 13 patients with asymmetric septal hypertrophic cardiomyopathy (HCM) including 2 patients without LGE and 11 with LGE (mean age 41 ± 13 years, 62% male) and 11 normal control subjects (mean age 52 ± 12years, 36% male, without cardiac disease or hypertension). MRI included cine imaging, LGE imaging, and a validated Look-Locker gradient echo cine IR technique for quantification of R1 in parallel short-axis locations. The myocardial partition coefficient was estimated by least-squares linear regression of R1 in myocardium against R1 in blood. MECVF was obtained by adjusting the partition coefficient by the patient’s hematocrit. We used a 18-segment model to quantify regional diffuse fibrosis and excluded segments with visible LGE in fibrosis quantification.

## Results

Patients with APH had a trend toward slightly higher MECVF as compared with normal controls at the base (0.30 vs. 0.27; Wilcoxon rank sum P = 0.09) and mid myocardial segments. In apical segments, there was a significant and substantial MECVF (0.38 vs. 0.29; P = 0.0002) in patients with APH compared to normal controls. There were no differences in MECVF between patients with APH and HCM in any segments.

## Conclusions

In patients with APH, MECVF is significantly elevated in the regions of apical segments despite no visualized LGE. These results suggest that myocardial R1 quantification can be used to characterize diffuse myocardial fibrosis at the left ventricular apex in patients with APH, and may provide an important tool to risk stratify these patients for adverse clinical outcome beyond traditional assessment of hypertrophy and LGE.

## Funding

None.

**Figure 1:**